# Can the pyruvate: ferredoxin oxidoreductase (*PFOR*) gene be used as an additional marker to discriminate among *Blastocystis* strains or subtypes?

**DOI:** 10.1186/s13071-018-3141-9

**Published:** 2018-10-29

**Authors:** Patricia Alarcon-Valdes, Guiehdani Villalobos, Williams Arony Martinez-Flores, Eduardo Lopez-Escamilla, Nelly Raquel Gonzalez-Arenas, Mirza Romero-Valdovinos, Fernando Martinez-Hernandez, Jonnathan Guadalupe Santillan-Benitez, Pablo Maravilla

**Affiliations:** 1 Facultad de Quimica, Universidad Autonoma del Estado de Mexico (UAEMex), Paseo Colon esq. Paseo Tollocan, Toluca, Estado de Mexico Mexico; 2 Instituto de Ecologia, Universidad Nacional Autonoma de Mexico (UNAM), 04510, Ciudad de Mexico, Ciudad de Mexico, Mexico; 3 Hospital General “Dr. Manuel Gea Gonzalez”, Calzada de Tlalpan 4800, Ciudad de Mexico, 14080 Ciudad de Mexico, Mexico

**Keywords:** *Blastocystis* spp., *Blastocystis* subtypes, Genetic polymorphism, Intestinal parasites, Pyruvate:ferredoxin oxidoreductase

## Abstract

**Background:**

*Blastocystis* spp. are the most prevalent intestinal eukaryotes identified in humans, with at least 17 genetic subtypes (ST) based on genes coding for the small-subunit ribosomal RNA (18S). It has been argued that the *18S* gene should not be the marker of choice to discriminate between STs of these strains because this marker exhibits high intra-genomic polymorphism. By contrast, pyruvate:ferredoxin oxidoreductase (PFOR) is a relevant enzyme involved in the core energy metabolism of many anaerobic microorganisms such as *Blastocystis*, which, in other protozoa, shows more polymorphisms than the *18S* gene and thus may offer finer discrimination when trying to identify *Blastocystis* ST. Therefore, the objective of the present study was to assess the suitability of the *PFOR* gene as an additional marker to discriminate among *Blastocystis* strains or subtypes from symptomatic carrier children.

**Methods:**

Faecal samples from 192 children with gastrointestinal symptoms from the State of Mexico were submitted for coprological study. Twenty-one of these samples were positive only for *Blastocystis* spp.; these samples were analysed by PCR sequencing of regions of the *18S* and *PFOR* genes. The amplicons were purified and sequenced; afterwards, both markers were assessed for genetic diversity.

**Results:**

The *18S* analysis showed the following frequencies of *Blastocystis* subtypes: ST3 = 43%; ST1 = 38%; ST2 = 14%; and ST7 = 5%. Additionally, using subtype-specific primer sets, two samples showed mixed *Blastocystis* ST1 and ST2 infection. For *PFOR*, Bayesian inference revealed the presence of three clades (I-III); two of them grouped different ST samples, and one grouped six samples of ST3 (III). Nucleotide diversity (π) and haplotype polymorphism (θ) for the *18S* analysis were similar for ST1 and ST2 (π = ~0.025 and θ = ~0.036); remarkably, ST3 showed almost 10-fold lower values. For *PFOR*, a similar trend was found: clade I and II had π = ~0.05 and θ = ~0.05, whereas for clade III, the values were almost 6-fold lower.

**Conclusions:**

Although the fragment of the *PFOR* gene analysed in the present study did not allow discrimination between *Blastocystis* STs, this marker grouped the samples in three clades with strengthened support, suggesting that *PFOR* may be under different selective pressures and evolutionary histories than the *18S* gene. Interestingly, the ST3 sequences showed lower variability with probable purifying selection in both markers, meaning that evolutionary forces drive differential processes among *Blastocystis* STs.

## Background

*Blastocystis* spp. are the most prevalent intestinal eukaryotes identified in humans and are one of the two known stramenopiles that can infect humans [1–4]. Low host specificity and extensive morphological and genetic diversity have been documented in this genus [4, 5]. Four stages or morphotypes are presently recognized in *Blastocystis*: vacuolar, also named “central body”, granular, amoeboid and cyst. Furthermore, 17 ribosomal lineages, known as subtypes (ST), have been described based on genotyping of the small-subunit ribosomal RNA (18S). ST1-ST9 are found in humans; however, they have also been reported in other hosts [5–8]. Some epidemiological and molecular data support a potential pathogenic role for these microorganisms [9–11]. However, the clinical relevance of *Blastocystis* is still controversial [12, 13]. Previous studies suggest that due to the exceptional inter- and intra-subtype genetic variability, it is not possible to establish, without doubt, the pathogenic role of *Blastocystis* because pathogenesis may be subtype-dependent [14, 15].

Recently, some factors known as “moonlighting proteins” were shown to be capable of enhancing virulence in eukaryotic pathogens; these proteins are enzymes with key metabolic functions in glycolysis, the pentose phosphate cycle or other fundamental intracellular processes. These proteins may perform non-catalytic roles with different functions depending on their cellular localization and the concentration of substrates or additional ligands. This group of proteins includes the pyruvate:ferredoxin oxidoreductase enzyme (PFOR) [16, 17].

PFOR is a Fe-S enzyme that uses thiamine pyrophosphate (TPP) and magnesium (Mg^+2^) as cofactors. It is involved in the energy metabolism of many anaerobic organisms and allows energy conservation by substrate-level phosphorylation with reversible catalysis of the oxidative decarboxylation of pyruvate to Acetyl-CoA and CO_2_. The resulting electrons are transferred to a low-redox potential, which depending on the physiological electron acceptor may involve hydrogen or activate molecules [18–20].

PFOR was initially identified in *Clostridium acidi-urici* [21], but the first description of its enzyme activity in eukaryotes was in *Entamoeba histolytica* [22]. It was subsequently described in other anaerobic parasites such as *Trichomonas vaginalis* [23], *Giardia lamblia* [24] and *Blastocystis* spp. [25]. *In vivo* and *in vitro* studies of the role of PFOR expression in parasites have suggested that it could be involved in cytoadherence, in the proliferation of trophozoites, and, under specific conditions, in the formation of subcutaneous abscesses [26]. Therefore, the purpose of this study was to assess the suitability of using the *PFOR* gene as an additional marker to discriminate among *Blastocystis* strains or subtypes from symptomatic carrier children.

## Methods

Faecal samples from 192 children who attended medical consultation for gastrointestinal disorders at the Hospital para el Niño del Instituto Materno Infantil from the State of Mexico (IMIEM) between January and June 2017 were analysed by coprological methods. Faust’s technique and microscopic observation were used to search for parasitic structures and to define the parasitic load per field using the 40× objective.

Approximately 50 mg of faeces from each participant was cultured in 7 ml of Boeck-Drbohlav modified medium at 37 °C for 3 days [27]. The concentration of *Blastocystis* cells was measured in a Neubauer chamber at 0 h, 48 h and 72 h. Additionally, an aliquot of up to 200 μl containing *Blastocystis* cells was used to extract DNA using a ZR Fecal DNA MiniPrepTM kit (Zymo Research, Irvine, CA, USA) according to the manufacturer’s protocol; the DNA concentration was determined by UV spectrophotometry, and DNA aliquots were stored at -20 °C until molecular analysis.

Subtype identification was performed according to Santin et al. [28]. To establish mixed infections between *Blastocystis* ST1, ST2 and ST3, ST-specific primers from previous reports were used [10, 11, 29–33]. To analyse the *PFOR* gene, specific primers for *Blastocystis* were designed based on available sequences in the GenBank database (ST7, XM_013038360; ST7, XM_013042447; ST4, XM_014671717; ST4, XM_014673113; ST7, XM_013039547; ST7, XM 013041057; ST7, XM_013038149; ST7, XM_013041791; and NandII ST1, EF512300). A suitable region of ~871 bp was chosen for amplification by the primers BlasPFOR-F: 5'-TGG CGA ACG CGA TGG GCT GCT CG-3' and BlasPFOR-R: 5'-CCA GCT GGA ACG GGT TCT CGC CC-3'.

The PCR mixture contained 25 pmol/μl each primer, 200 ng/μl genomic DNA, 2 mM MgCl_2_, 1× PCR buffer (200 mM Tris-HCl, pH 8.4, 500 mM KCl), 0.2 mM dNTPs, 0.01 mg of BSA and 1 U of Taq DNA polymerase (Invitrogen, Carlsbad, CA, USA); concentration of reagents was calculated and adjusted for 25 μl volume reaction.

A total of 40 cycles, each consisting of 94 °C for 30 s, 69 °C for 90 s and 72 °C for 60 s, was performed; an initial pre-heat step at 94 °C for 5 min and a final extension step at 72 °C for 7 min were also included. The PCR products were separated by 1.2% agarose gel electrophoresis, visualized by ethidium bromide staining (0.5 μg/ml) and purified with an illustra^TM^ GFX^TM^ PCR DNA and Gel Band Purification kit (GE Healthcare, Little Chalfont, Buckinghamshire, UK). The purified products were sequenced in both directions at the Instituto de Biologia, Universidad Nacional Autonoma de Mexico. DNA of the *Blastocystis* strain ATCC-50754 (ST3) was used as a positive control.

All sequences were subjected to BLAST searches in the GenBank database to confirm they were from *Blastocystis* spp. specimens. Sequences for both genes obtained in this study were aligned with those available in public databases using the Clustal W and Muscle algorithms included in MEGA software version 7.0.26 [34–36]. Phylogenetic reconstruction was conducted using a Bayesian approach with MrBayes version 3.2 [37]. The analysis was performed for 10,000,000 generations with sampling trees every 100 generations. Trees with scores lower than those at the stationary phase (‘burn-in’) were discarded, and trees that reached the stationary phase were collected and used to build majority consensus trees. Other sequences of *18S* from different *Blastocystis* STs and *PFOR* from other pathogens (such as *Entamoeba* spp. and *Trichomonas vaginalis*) were obtained from GenBank and used as references.

Genetic diversity indices for both the *18S* and *PFOR* sequences were obtained with DnaSPv6 software [38] and included nucleotide diversity (π, the average proportion of nucleotide differences between all possible pairs of sequences in the sample) and haplotype polymorphism (θ, the proportion of nucleotide sites that are expected to be polymorphic in any suitable sample from this region of the genome). These indices range in value from 0 to 1 and are used to assess polymorphisms at the DNA level, to measure variability within or between ecological populations, and to examine the genetic variability in related species or their evolutionary relationships. Additionally, to assess if our sequences were evolving randomly (neutrally) or were under a selection process, they were subjected to Tajima’s D test, in which positive values indicate a decrease in population size or balancing selection, while negative values suggest expansion of the population or purifying selection [39].

## Results

In the 192 samples analysed by microscopy, the following parasites were identified: *Blastocystis* spp. (36.5%); *Entamoeba coli* (33%); *Endolimax nana* (32%); *Entamoeba histolytica*/*E. dispar* (15%); *Hymenolepis nana* (10%); and *Enterobius vermicularis* (5.5%). Samples belonging to 21 children exhibited *Blastocystis* single infections (Table 1). In these cases, abdominal pain was the main symptom described by all patients, and the vacuolar form was observed in all samples. The clinical and demographic data, parasite load, and genotyping of both the *18S* and *PFOR* genes are summarized in Table 1.

**Table 1:** Demographic, clinical and parasitological data, *Blastocystis* subtypes by *18S* rDNA and clades by *PFOR*

Sample	Gender^a^/age^b^	Symptoms	Parasite load (CPS-40×)	Stage-morphotype culture (48 h)	Subtypes (*18S* rDNA)		Clades (*PFOR*)
					Santin primers set	subtype-specific primers set	
01	M/4	Abdominal pain	> 5	Vacuolar	ST1 (MH453913)	ST1	I (MH507339)
02	F/11	Abdominal pain	> 5	Vacuolar	ST3 (MH453914)	ST3	I (MH507340)
03	F/13	Abdominal pain and diarrhoea	> 10	Vacuolar, granular, amoeboid	ST3 (MH453915)	ST3	III (MH507341)
04	M/7	Abdominal pain	> 5	Vacuolar	ST2 (MH453916)	ST2	II (MH507342)
06	M/14	Abdominal pain	> 5	Vacuolar	ST1 (MH453917)	ST1, ST2	II (MH507343)
07	F/10	Abdominal pain	> 5	Vacuolar	ST2 (MH453918)	ST2	II (MH507344)
08	F/9	Abdominal pain	> 5	Vacuolar	ST3 (MH453919)	ST3	III (MH507345)
09	F/7	Abdominal pain	> 5	Vacuolar	ST3 (MH453920)	ST3	I (MH507346)
11	M/13	Abdominal pain	> 5	Vacuolar	ST3 (MH453921)	ST3	III (MH507347)
14	M/3	Abdominal pain and diarrhoea	> 10	Vacuolar, granular, amoeboid	ST3 (MH453922)	ST3	I (MH507348)
15	M/7	Abdominal pain and mucous stool	> 10	Vacuolar, granular	ST3 (MH453923)	ST3	III (MH507349)
21	F/1	Abdominal pain	> 5	Vacuolar	ST1 (MH453924)	ST1	I (MH507350)
22	M/14	Abdominal pain	> 10	Vacuolar, granular	ST1 (MH453925)	ST1	I (MH507351)
24	M/6	Abdominal pain	> 5	Vacuolar	ST7 (MH453926)	ND^c^	I (MH507352)
25	F/10	Abdominal pain	> 5	Vacuolar	ST1 (MH453927)	ST1, ST2	I (MH507353)
33	M/8	Abdominal pain and diarrhoea	> 5	Vacuolar	ST3 (MH453928)	ST3	III (MH507354)
35	F/8	Abdominal pain and diarrhoea	> 5	Vacuolar	ST1 (MH453929)	ST1	I (MH507355)
102	F/8	Abdominal pain and diarrhoea	> 10	Vacuolar	ST3 (MH453930)	ST3	III (MH507356)
37	F/9	Abdominal pain	> 10	Vacuolar	ST2 (MH453931)	ST2	II (MH507357)
45	M/3	Abdominal pain	> 5	Vacuolar	ST1 (MH453932)	ST1	I (MH507358)
46	M/5	Abdominal pain and diarrhoea	> 5	Vacuolar	ST1 (MH453933)	ST1	I (MH507359)
ATCC-50754	–	–	–	–	ST3 (MH453934)	–	III (MH507360)

^a^*F*, female, *M*, male

^b^Age in years

^c^ *ND*, Not determined for ST7, but ST1, ST2 and ST3 were tested in this sample

For the 21 samples positive for *Blastocystis,* as well as for the commercial strain ATCC-50754, all sequences were obtained for both the *18S* and *PFOR* genes (GenBank: MH453913-MH453934 and MH507339-MH507360, respectively). In this study, the *Blastocystis* STs were identified as ST3 (43%), ST1 (38%), ST2 (14%) and ST7 (5%); only two samples showed mixed ST infection with ST1 and ST2.

**Fig. 1:**
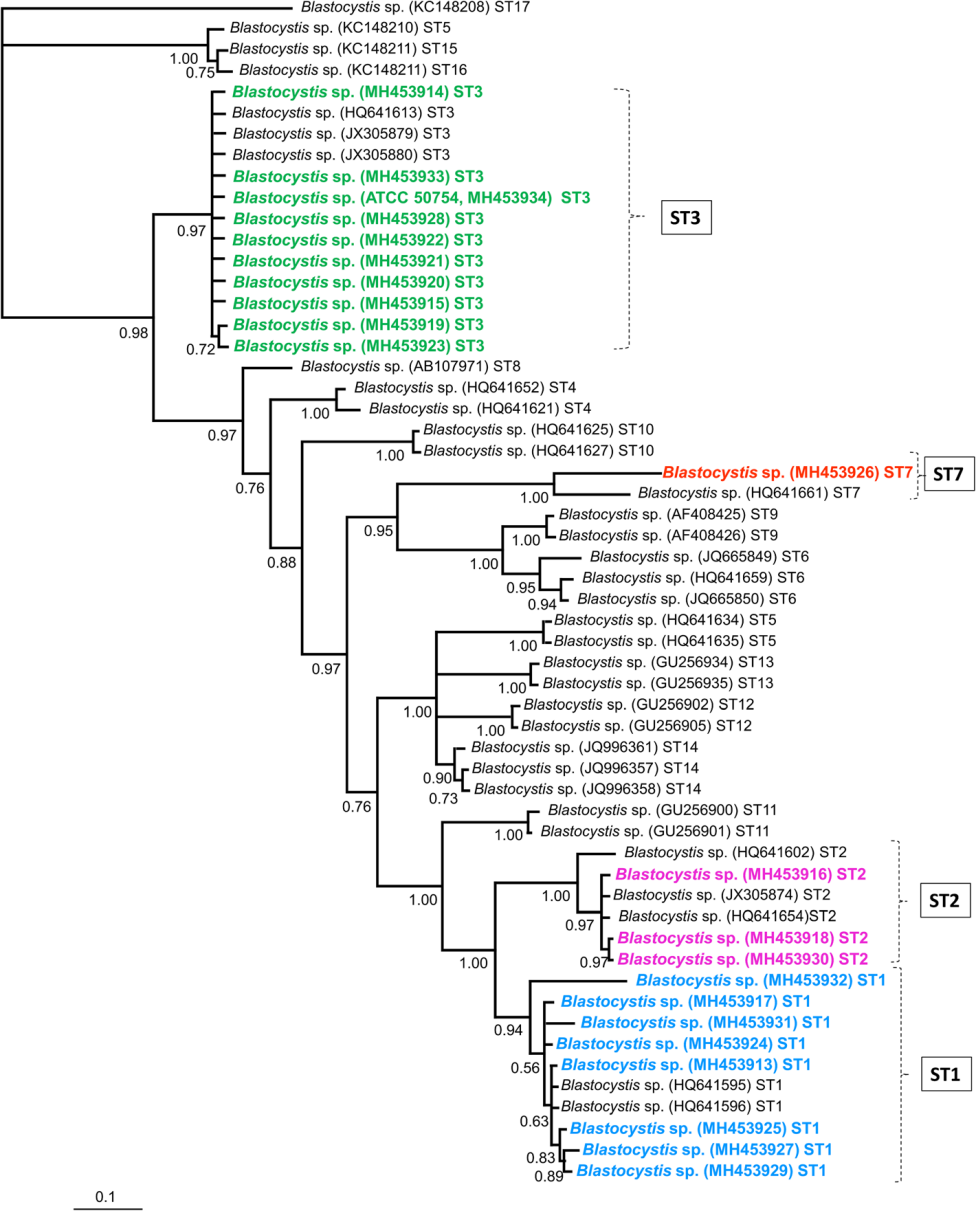
Bayesian phylogenetic tree for the *Blastocystis* sequences obtained from children from Mexico using a fragment of the *18S* rRNA gene. The values of the nodes indicate posterior probabilities using 10,000,000 generations. The GenBank accession numbers of the reference sequences are included; newly sequenced isolates are shown in different colours, ST1 is blue, ST2 is pink, ST3 is green and ST7 is red

**Fig. 2:**
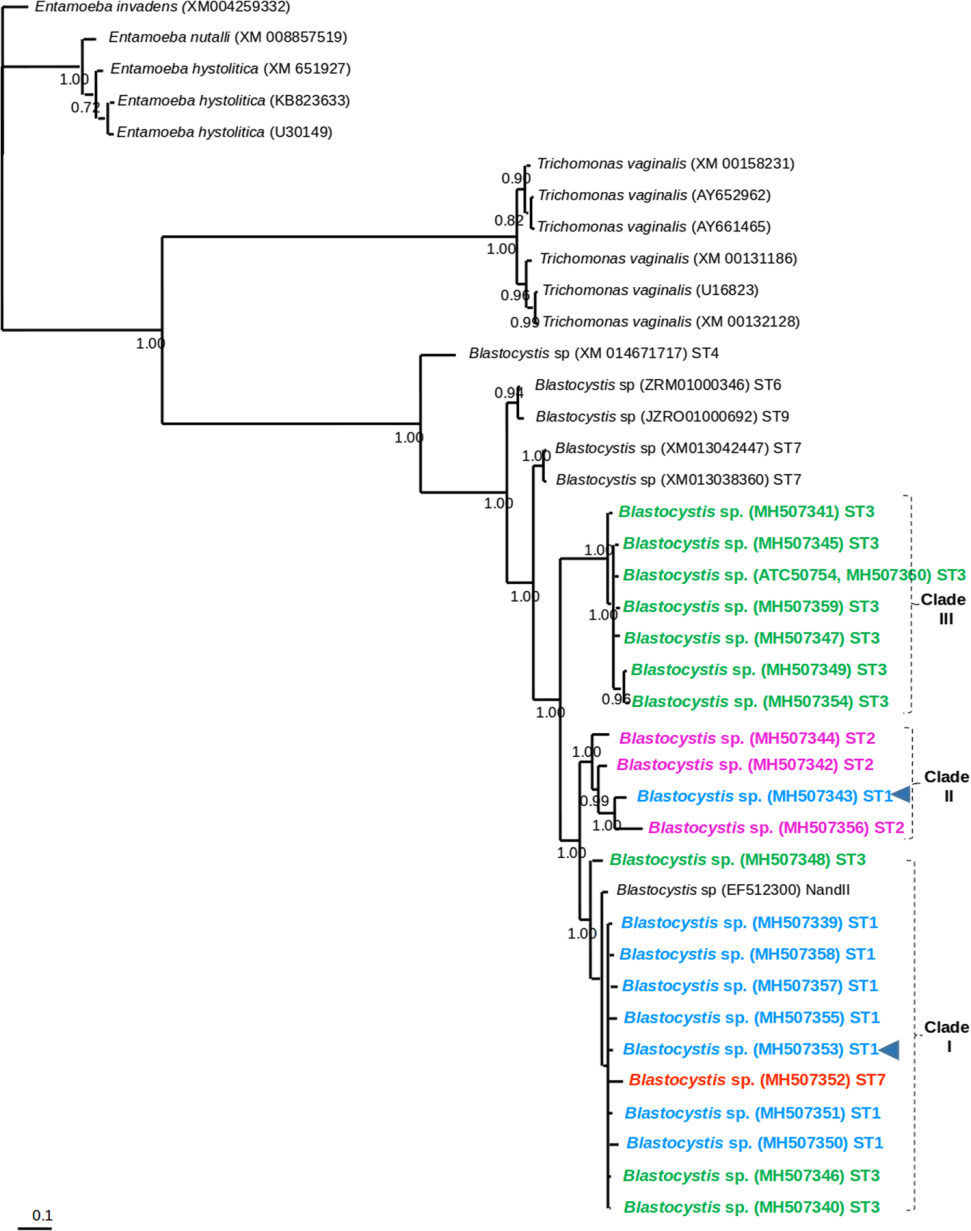
Bayesian phylogenetic tree for the PFOR *Blastocystis* sequences obtained from children from Mexico using a fragment of the *PFOR* gene. The values of the nodes indicate posterior probabilities using 10,000,000 generations. The GenBank accession numbers of the reference sequences are included; newly sequenced isolates are shown in different colours, ST1 is blue, ST2 is pink, ST3 is green and ST7 is red. Arrowheads point out isolates with mixed infections of ST1 with ST2

The Bayesian phylogenetic tree built for *18S* corroborated the *Blastocystis* ST distribution (Fig. 1). In contrast, the tree generated for *PFOR* grouped parasite species into different clades, and the main *Blastocystis* clade did not show a clear distribution of STs. In two clades (I and II), a mixture of STs was observed; in clade III, six ST3 samples plus the ATCC commercial strain were grouped (Fig. 2).

The genetic diversity indices obtained in the *18S* analysis showed similar values between ST1 and ST2, with π = 0.025 and θ = 0.036. Recall that π denotes the proportion of nucleotide differences between possible pairs of sequences and θ is the proportion of nucleotide sites that are expected to be polymorphic in any suitable sample from this region of the genome. Remarkably, the values for ST3 were almost 10-fold lower than other STs (π = 0.004 and θ = 0.005). In the same analysis of the *PFOR* gene, a similar trend was found for clades I and II (π = 0.05 and θ = 0.05); whereas for clade III, the genetic diversity indices values were π = 0.008 and θ = 0.009. Tajima’s D test showed negative values for ST1-ST3 and clades I-III for both the *18S* and *PFOR* markers (Table 2).

**Table 2:** Genetic polymorphism indexes between different *Blastocystis* sequences

Marker	No. of sequences	H^a^	Hd^b^	π^c^ ± SD^d^	θ^e^ ± SD	Tajima’s D (*P*-value)
*18S* rDNA						
ST1	10	6	0.867	0.0293 ± 0.0157	0.0470 ± 0.0199	-1.9031 (≤ 0.05)
ST2	6	5	0.933	0.0201 ± 0.0109	0.0258 ± 0.0129	-1.3898 (≥ 0.10)
ST3	13	8	0.894	0.0045 ± 0.001	0.0055 ± 0.0028	-0.6024 (≥ 0.10)
*PFOR*						
Clade I	10	8	0.956	0.0311 ± 0.0117	0.0457 ± 0.0199	-1.7406 (≤ 0.05)
Clade II	4	4	1.000	0.0655 ± 0.0137	0.0635 ± 0.0350	-0.5006 (≥ 0.10)
Clade III	6	5	0.933	0.0079 ± 0.0027	0.0098 ± 0.0052	-1.2217 (≥ 0.10)

^a^H, number of haplotypes

^b^Hd, haplotype diversity

^c^π, nucleotide diversity

^d^SD, standard deviation

^e^θ, haplotype polymorphism

## Discussion

It has been argued that the *18S* rRNA gene, which is commonly used to distinguish *Blastocystis* STs, should not be the marker of choice for discriminating between strains within these STs [40, 41]. Poirier et al. [40] reported that although *Blastocystis* has a high genetic diversity, the *18S* rRNA gene possesses at least 17 copies that can be grouped into 6 clades. However, in ST7 comparisons with different strains, 4 of the 6 clades showed high identity within the strains compared. Markers other than the *18S* rRNA gene have been used to distinguish among *Blastocystis* strains or subtypes [40–42]. Villalobos et al. [41] compared the internal transcribed spacers (ITS) of ST1, ST2, ST3 and ST7 identified in human samples and found two variants of ST1. Poirier et al. [40] reported that a single-copy subtyping rDNA marker in the genome of mitochondria-like organelles was capable of successfully subtyping 66 isolates of *Blastocystis* ST1-ST10 from both humans and animals and could also detect co-infections by different isolates of the same ST. In the present study, we assessed the level of genetic diversity in an ~871 bp region of the *PFOR* gene of *Blastocystis* isolates from symptomatic carriers.

*Blastocystis* subtyping in samples provided by carriers from the State of Mexico has not been previously documented. We found that ST3 and ST1 were the most frequent subtypes, consistent with previous reports describing children infected with *Blastocystis* from other states in Mexico [41, 43]. Similarly, the values obtained in this study for nucleotide diversity (π) and haplotype polymorphism (θ) for the *18S* gene were in accordance with previous studies of genetic diversity in *Blastocystis* infections in children from other geographical regions of Mexico [41, 43]. Interestingly, the π and θ values for ST1 or ST2 were almost 10-fold higher than those for ST3, indicating a high reduction of the variability within and among sequences in this subtype. This result is consistent with previous studies in which isolates of ST3 from patients with irritable bowel syndrome showed lower genetic variability than those from asymptomatic carriers [44]. A study focused on the genetic variability and host specificity of *Blastocystis* spp. in wild howler monkeys from two rainforest areas in the south-eastern region of Mexico reported that ST1 exhibits a generalist profile similar to a metapopulation, whereas ST2 existed as a set of local populations [5]. Another study aimed to determine the frequency and distribution of *Blastocystis* subtypes in free-ranging *Macaca fascicularis* in Thailand and showed that ST3 was the most common subtype detected (36%), followed by ST2 and ST1 (24% and 17%, respectively). However, some new subtype alleles were also identified [45]. These reports suggest that the presence of different levels of cryptic host specificity in *Blastocystis* may modify the genetic population structure of this microorganism, including its levels of genetic variability. Additionally, the negative values of Tajima’s D test suggest a recent expansion process or an effect of purifying selection in ST1-ST3 [39], strengthening the action of other evolutionary forces in the epidemiological landscape of *Blastocystis*. The mixed infections with ST1 and ST2 identified in two samples in the present study are in accordance with other studies reported mixed infections of ST1 with other STs (*c.*10%) [46, 47].

Regarding the phylogenetic tree for *PFOR*, sequences that belonged to other parasites were grouped into separate clades as expected. The *PFOR* sequences that belonged to *Blastocystis* were grouped into three clades. In two of the clades, different STs were gathered without a predominant ST; only one clade grouped seven samples of ST3 and also included the ATCC-50754 strain (ST3). The presence of differences between the *PFOR* and *18S* trees is not surprising and is common when phylogenetic inferences drawn from different genes are compared [48]. Therefore, this initial analysis, although indicating that the *PFOR* gene locus used in the present study is not sensitive enough to differentiate subtypes, suggests that the phylogeny of *PFOR* may provide inferences about the function of the protein instead of the relationship of the group. On the other hand, it has been argued that in some genomes of intestinal pathogens [49], including *Blastocystis* [50], *PFOR* is a single-copy gene, and hence this marker may be subjected to different selection pressures, according to studies of multi- and single-copy genes [39]. In addition, the results obtained could have been influenced by other evolutionary processes, such as homoplasy [51], genetic hitchhiking [52] or simply the high conservation of the analysed *PFOR* fragment, which corresponds to a region inside the active site of the protein. To clarify these factors, complete sequencing of the *PFOR* gene should be performed. When comparing genetic resolution to the *18S* gene, future studies for of the *PFOR* gene and new genetic molecular markers must address mixed infections to avoid problematic clustering, such as the clustering of clades I and II observed in this study. *18S* gene analysis has shown that this marker is sensitive enough to resolve phylogenetic relationships, population differentiation events and cryptic infections in *Blastocystis* [41, 43, 53–55]. Finally, the knowledge of the genetic variation within and between populations can be applied to the epidemiology and the control of parasites because these biological features influence future evolutionary changes, genetic differentiation, and speciation in many pathogens [5, 40, 41].

## Conclusions

Although the fragment of the *PFOR* gene analysed in present study did not allow discrimination between *Blastocystis* STs, this marker grouped the samples in three strongly-supported clades, suggesting that *PFOR* may be under different selective pressures and evolutionary histories than the *18S* gene. Interestingly, ST3 sequences showed lower variability with probable purifying selection in both markers, meaning that evolutionary forces are driving differential processes among the *Blastocystis* STs. Finally, according to Poirier et al. [56], the controversial role of *Blastocystis* spp. as pathogens remains unclear. Thus, there is still a need to conduct epidemiological studies focused on distinguishing between strains within subtypes of this genus.

## Abbreviations

*18S* rDNA: Small-subunit ribosomal RNA
ATCC: American Type Culture Collection
BSA: Bovine serum albumin
CoA: Coenzyme A
Fd: Ferredoxin
Fe-S: Iron-Sulfur
H: Number of haplotypes
Hd: Haplotype diversity
IMIEM: Instituto Materno Infantil from the State of Mexico
PCR: Polymerase chain reaction
PFOR: Pyruvate:ferredoxin oxidoreductase enzyme
SD: Standard deviation
ST: Subtype
TPP: Thiamine pyrophosphate
θ: Haplotype polymorphism
π: Nucleotide diversity
